# Mannooligosaccharides modulate mucin O-glycan–gut microbe interactions and preserve the intestinal barrier in weaned piglets

**DOI:** 10.1186/s40104-026-01492-x

**Published:** 2026-08-24

**Authors:** Shanghang Liu, Nian Liu, Yutao Qiu, Hao Li, Fan Zhang, Qian Jiang, Jing Wang, Yulong Yin, Bie Tan, Xiaokang Ma

**Affiliations:** 1 College of Animal Science and Technology, Hunan Agricultural University, Changsha, 410128 China; 2 Institute of Subtropical Agriculture, Chinese Academy of Sciences, Changsha, 410125 China

**Keywords:** Glycosylation, Intestinal damage, Intestinal microbiota, Mannooligosaccharides, O-glycans, Weaned piglets

## Abstract

**Background:**

The intestinal microbiota and mucosal barrier are closely intertwined, with mucin O-glycans playing a key role in maintaining intestinal homeostasis. However, how nutrition regulates mucin O-glycans remains poorly understood. This study investigated the regulatory effects of mannooligosaccharides (MOS) on mucin secretion and O-glycan modification in a weaned piglet model. We evaluated the effects of MOS on intestinal morphology, gut microbiota, mucin secretion, and O-glycosylation profiles in weaned piglets.

**Results:**

Transcriptome analysis revealed that MOS influenced key KEGG pathways, including mucin O-glycan biosynthesis, through the regulation of genes such as *GALNT*, *GCNT3*, and *POFUT1*. Furthermore, MOS increased the proportion of Core 2 structures in mucin and modulated the proportion of long-chain, sulfated and sialylated O-glycans, which may help protect the mucus layer from microbial degradation. Moreover, MOS improved colonic microbiota composition by promoting beneficial *Lactobacillus* species and suppressing potentially pathogenic *Escherichia coli*.

**Conclusion:**

This study highlights the molecular effects of MOS on mucin synthesis and modification, which may help preserve intestinal microbiota balance. These effects are important for maintaining intestinal barrier function in weaned piglets.

**Supplementary Information:**

The online version contains supplementary material available at 10.1186/s40104-026-01492-x.

## Background

Inflammatory bowel disease (IBD), including ulcerative colitis and Crohn’s disease, is a chronic systemic inflammatory disease that primarily affects the gastrointestinal tract [1]. Its pathogenesis is closely related to mucin secretion. Patients with IBD exhibit obvious structural abnormalities in the intestinal mucosa, resulting in alterations in mucin secretion, core structure, and sulfation or sialylation of oligosaccharide residues [2]. Intestinal mucin, which is secreted by goblet cells, is a fundamental component of the intestinal mucus barrier and constitutes the primary innate defense against intestinal pathogenic bacteria and exogenous harmful substances [3]. Beyond physical protection, intestinal mucin plays a pivotal role in immune responses, including antigen presentation, mitigation of external stimuli, and regulation of bacterial colonization within the gastrointestinal tract.

The integrity of the intestinal mucus layer is closely linked to the balance of the intestinal microbial ecosystem [4–6]. Mucins consist of homologous oligomers with an N-terminal oxidized glycosylation domain and a C-terminal cysteine-rich domain. Their protein structure is heavily modified by O-glycans, and changes in O-glycans are of paramount importance. Alterations in O-glycans, which account for 80% of mucin composition, can lead to intestinal barrier dysfunction and impaired mucosal integrity [7, 8]. Previous studies have reported that MUC2 secretion by goblet cells is significantly increased in patients with severe Crohn’s disease and is accompanied by increased mucin sulfation. Mucin overexpression and abnormal glycosylation are the major features of Crohn’s disease [9, 10]. Structural changes in mucin have also been found in the gut of patients with ulcerative colitis, mainly manifested as reduced mucin glycosylation and shortened oligosaccharide side chains [9]. These findings collectively underscore the critical role of mucin secretion and O-glycosylation in the pathogenesis of IBD. Notably, nutritional regulation in this context remains poorly understood, highlighting the need for further investigation.

The human gastrointestinal tract harbors a complex microbiome and provides a nutrient-rich luminal environment, both of which are essential for maintaining overall health [11]. Numerous studies have confirmed the importance of functional carbohydrates, including oligosaccharides and various natural plant polysaccharides, in promoting intestinal health in animals. Chronic deficiency of microbiota-accessible carbohydrates in the gut can compromise the intestinal barrier integrity [12]. However, the effects of functional carbohydrates on the interaction between mucin O-glycans and intestinal microbes remain insufficiently understood, and functional gene screening remains limited. MOS (mannooligosaccharides) represents a class of functional polysaccharides and non-digestible oligosaccharides. These compounds are primarily composed of 2–10 mannose or glucose molecules linked by α-(1,2), α-(1,3), and α-(1,6) glucosidic bonds. MOS exhibits dual immunoregulatory properties and can adsorb intestinal pathogens. Yeast-derived MOS is a phosphorylated glucomannan-protein complex extracted by enzymolysis from mannan-rich yeast cell walls [13, 14].

MOS serves as an ideal substrate for beneficial gut bacteria, including *Bifidobacterium* spp. and *Bacteroides* spp. These bacteria can ferment MOS into short-chain fatty acids (SCFAs), creating an environment conducive to the growth of beneficial intestinal flora [15]. In addition, mannose residues in MOS can competitively bind to mannose-lectin receptors on the surface of Gram-negative pathogens, inhibit the colonization and adhesion of pathogens such as *Salmonella* and *Escherichia coli*, promote the intestinal elimination of pathogens, and optimize the intestinal microbiota structure of piglets [16]. MOS therefore plays a pivotal role in regulating intestinal microecology. Previous studies have indicated that MOS has the potential to influence intestinal mucin secretion. Probiotic proliferation in the presence of MOS can upregulate mucin gene expression and enhance mucin secretion in goblet cells [17]. Furthermore, MOS has been shown to mitigate intestinal mucosal injury and contribute to the maintenance of intestinal barrier integrity and epithelial function. However, whether MOS participates in mucin synthesis and modification remains to be further investigated.

Pigs share many similarities with humans in intestinal digestive physiology, immunity, and other functions. Consequently, they serve as an alternative model for human diseases, owing to their similar clinical manifestations and susceptibility to many human intestinal pathogens [18]. Because of their low intestinal barrier function and immunity, weaned piglets are a natural model of intestinal stress syndrome and inflammatory diseases. In light of these advantages, this study combined liquid chromatography-tandem mass spectrometry (LC–MS/MS) with transcriptomics to investigate the regulatory effects of MOS on intestinal mucin synthesis and glycosylation in weaned piglets. These findings may provide insights into the potential application of MOS in drug development for intestinal stress syndrome and inflammatory diseases, and lay a foundation for the utilization of functional carbohydrates to improve gut health and host metabolism.

## Materials and methods

### Animals, diets, and experimental design

In this experiment, 24 healthy weaned castrated male piglets (Duroc × Landrace × Yorkshire, 28 days old) with similar body weight (BW, 7.8 ± 0.14 kg) were randomly assigned to 24 pens, with one pig per pen. All piglets had free access to feed and water. The pig houses were maintained according to routine procedures for pest control, immunization, cleaning, and disinfection.

In a randomized complete block design, piglets were blocked by initial BW, and pens were allocated to treatments in a balanced manner across the animal house to minimize environmental variation. Piglets were randomly divided into three dietary treatment groups: a basal diet (CON), a basal diet supplemented with antibiotics (75 mg/kg aureomycin plus 150 mg/kg quinolone; AB), and a basal diet supplemented with 2,000 mg/kg mannooligosaccharides (MOS), with 8 replicates per treatment. The AB group was included as a positive control to benchmark the effects of MOS against conventional antibiotic treatment in the context of antibiotic growth promoter replacement. The pre-experimental period lasted for 3 d, followed by a 14-day experimental period. and the basal diet was formulated according to NRC (2012) [19] and the nutritional requirements of weaned piglets (Table S1). The MOS used in this experiment was derived from yeast cell walls, and was a food-grade product with 90% purity and a degree of polymerization of 2–10 (#S51698, Shanghai Yuanye Bio-Technology Co., Ltd., Shanghai, China) [20]. During the formal trial, piglet BW and feed consumption were measured on d 1, 7, and 14 to assess growth performance. Diarrhea occurrence was monitored by visual assessment and evaluated according to the method of Huang et al. [21]. Each day at 8:00 and 16:00, piglets were individually examined for signs of fecal contamination, redness, and edema, and detailed records were maintained.

After the experiment, all pigs were slaughtered, and fresh intestinal samples were collected from each piglet. The jejunum, ileum, and colon were separated, and fixed in 4% paraformaldehyde for determining intestinal morphology, and then sampled for subsequent analyses. The colonic mucosa was carefully scraped from the underlying muscle layer using a sterile glass slide on an ice-chilled plate. Simultaneously, colonic mucosa and colonic content were collected, frozen in liquid nitrogen, and stored at −80 °C for SCFA and microbiota analyses.

### AB-PAS staining and immunofluorescence staining

Two paraffin sections were obtained from fixed colon samples. One was subjected to Alcian blue-periodic acid-Schiff (AB-PAS) staining to assess mucin secretion and the distribution of neutral and acidic mucins according to Xia et al. [22]. The other section was immunofluorescence-labeled with primary antibodies against MUC2, MUC5AC, and MUC5B to compare the expression and distribution of intestinal mucins in piglets after MOS treatment. Section images were independently evaluated by two researchers who were not involved in the experiment.

### Cytokine measurement

Colon tissue samples were homogenized in ice-cold phosphate-buffered saline (PBS) containing protease inhibitors. The homogenates were centrifuged at 12,000 × *g* for 10 min at 4 °C, and the supernatants were collected for cytokine analysis. Concentrations of interleukin-22 (IL-22), interferon-γ (IFN-γ), interleukin-1β (IL-1β), tumor necrosis factor-α (TNF-α), and interleukin-4 (IL-4) were determined using commercial porcine-specific enzyme-linked immunosorbent assay (ELISA) kits (Jiangsu Meimian Industrial Co., Ltd., Yancheng, Jiangsu, China) according to the manufacturer's protocols. The absorbance was read at 450 nm using a microplate reader (BioTek Instruments, Winooski, VT, USA), and cytokine concentrations were calculated from standard curves. All samples were assayed in duplicate, and results were normalized to total protein content, expressed as pg/mg protein.

### DNA extraction and 16S rRNA gene sequencing

Total DNA from ileal and colonic contents was extracted using the QIAamp Fast DNA Stool Mini Kit (Qiagen, Hilden, Germany). Total DNA was evaluated by 1% agarose gel electrophoresis, and DNA concentration and purity were determined using a Nanodrop 2000 spectrophotometer (Thermo Fisher Scientific, Wilmington, USA). The V3–V4 hypervariable region of the 16S rRNA gene was amplified using universal primers. The primer sequences were as follows: 338 F (5'-ACTCCTACGGGAGGAGCAG-3') and 806R (5'-GGACTACHVGGGTWTCTAAT-3'). Sequence data were analyzed using the Quantitative Into Microbial Ecology (QIIME) package (version 1.7.0) and R software (version 3.2.0), with the vegan and ggplot2 packages used for β-diversity analysis and visualization. OTU tables generated in QIIME, principal coordinate analysis (PCoA), and ANOSIM cluster analysis were used to calculate and evaluate the β-diversity. PCoA based on Bray–Curtis distances and linear discriminant analysis effect size (LEfSe) analysis were conducted using the Majorbio Cloud Platform (www.majorbio.com). Differential taxa between two groups were identified using the Mann–Whitney U test, with *P* < 0.05 and a LDA score threshold of 3.0.

### Transcriptome sequencing

TRIzol^®^ reagent (Invitrogen, Carlsbad, CA, USA) was used for RNA extraction, purification, and quality assessment. All RNA samples were sent to Majorbio Co., Ltd. (Shanghai, China) for mRNA isolation, cDNA library construction, and Illumina sequencing. Raw data were assembled and subjected to quality control, and the clean data obtained after filtering were used for subsequent analyses. Clean reads were aligned to the reference genome, followed by evaluation of sequencing coverage and depth. Gene expression levels were calculated using the reads per kilobase of transcript per million mapped reads (RPKM) method. Differentially expressed genes (DEGs) were selected based on fold change and significance level. The distribution of DEGs in Gene Ontology (GO) classifications was calculated to identify enriched GO terms and the biological processes involved in DEGs after MOS treatment. Kyoto Encyclopedia of Genes and Genomes (KEGG) pathway analysis of DEGs was performed using the Majorbio Cloud Platform.

### RNA extraction and quantitative real-time PCR

Colonic mucosa was placed in a centrifuge tube and fully lysed with TRIzol reagent. Chloroform, isopropyl alcohol, and diethyl pyrocarbonate (DEPC)-treated water were then used for RNA extraction and purification. RNA was reverse-transcribed into cDNA using the Evo M-MLV-RT Premix Kit. RT-qPCR was performed using the SYBR Green Pro Taq HS Premix PCR Kit. All kits were purchased from Accurate Biotechnology (Hunan) Co., Ltd. (Changsha, China). Primer information is shown in Table S2, and primers were synthesized by Sangon Biotech Co., Ltd. (Shanghai, China).

### Mucin isolation and purification and O-glycan release

Mucin was purified according to the methods of Venkatakrishnan et al. [23] and Bergstrom et al. [4]. Briefly, scraped colonic mucosa and protease inhibitor were placed together in a centrifuge tube. Five volumes of extraction buffer (6 mol/L GuCl, 0.1 mol/L Tris, and 1 mmol/L EDTA, pH = 8.0) were added, and the mixture was homogenized using a tissue grinder and then rotated gently for 5 h. The supernatant was collected after centrifugation (14,000 r/min, 10 min), and the precipitate was washed twice with 500 μL extraction buffer. The supernatants were then combined. Following extraction, the sample was reduced in a 37 °C water bath for 5 h with 100 mmol/L dithiothreitol (DTT). Subsequently, overnight treatment with 250 mmol/L iodoacetamide (IAA) dissolved the gel-forming mucin, and the sample was freeze-dried after dialysis. Purified mucin then underwent reduced β-elimination for 16 h at 50 °C using 0.5 mol/L sodium borohydride and 50 mmol/L sodium hydroxide to release the glycans. The released O-glycans were subsequently analyzed by LC–MS/MS (Q Exactive Plus, Thermo Fisher Scientific, Waltham, MA, USA).

### Quantitative analysis of short-chain fatty acids

Following the method of Xia et al. [22], we quantified SCFAs in colonic contents using high-performance gas chromatography (HPGC). Colonic contents stored at −80 °C were thawed, and 1 g of chyme from each weaned piglet was accurately weighed into a 10-mL centrifuge tube. After overnight dissolution in 5 mL ultrapure water, the mixture was centrifuged at 10,000 × *g* for 10 min at 4 °C. The resulting supernatant was mixed with 25% metaphosphoric acid solution at a ratio of 9:1, followed by further centrifugation and filtration through a 0.45-μm microporous filter membrane. Chromatographic separation and detector analysis were then performed using an Agilent 8890 gas chromatography (Agilent Technologies, USA) to determine SCFA contents, including acetate, propionate, and butyrate.

### Statistical analysis

Data were organized using Excel 2010 software and then subjected to statistical analyses including one-way ANOVA and the Wilcoxon rank‑sum test using SAS version 9.2 software. Spearman correlation analysis was used to investigate potential relationships between differential microbial results and transcriptomic DEG levels, as well as between mucin gene expression levels and relative concentrations of mucin oligosaccharides. For microbiota data, α-diversity indices (Chao1, Shannon, and Simpson) were compared among groups using the Kruskal–Wallis test followed by Dunn’s post hoc test. β-Diversity was evaluated using PCoA based on Bray–Curtis distances, and differences between groups were tested by PERMANOVA (Adonis) with 999 permutations. Differentially abundant taxa were identified using LEfSe (linear discriminant analysis effect size), with the threshold set at LDA score > 3.0 and *P* < 0.05. For all results, *P* < 0.05 was considered statistically significant, *P* < 0.01 was considered extremely significant, and 0.05 ≤ *P* < 0.10 was considered a trend.

## Results

### MOS alleviates diarrhea and growth retardation in weaned piglets

**Fig. 1:**
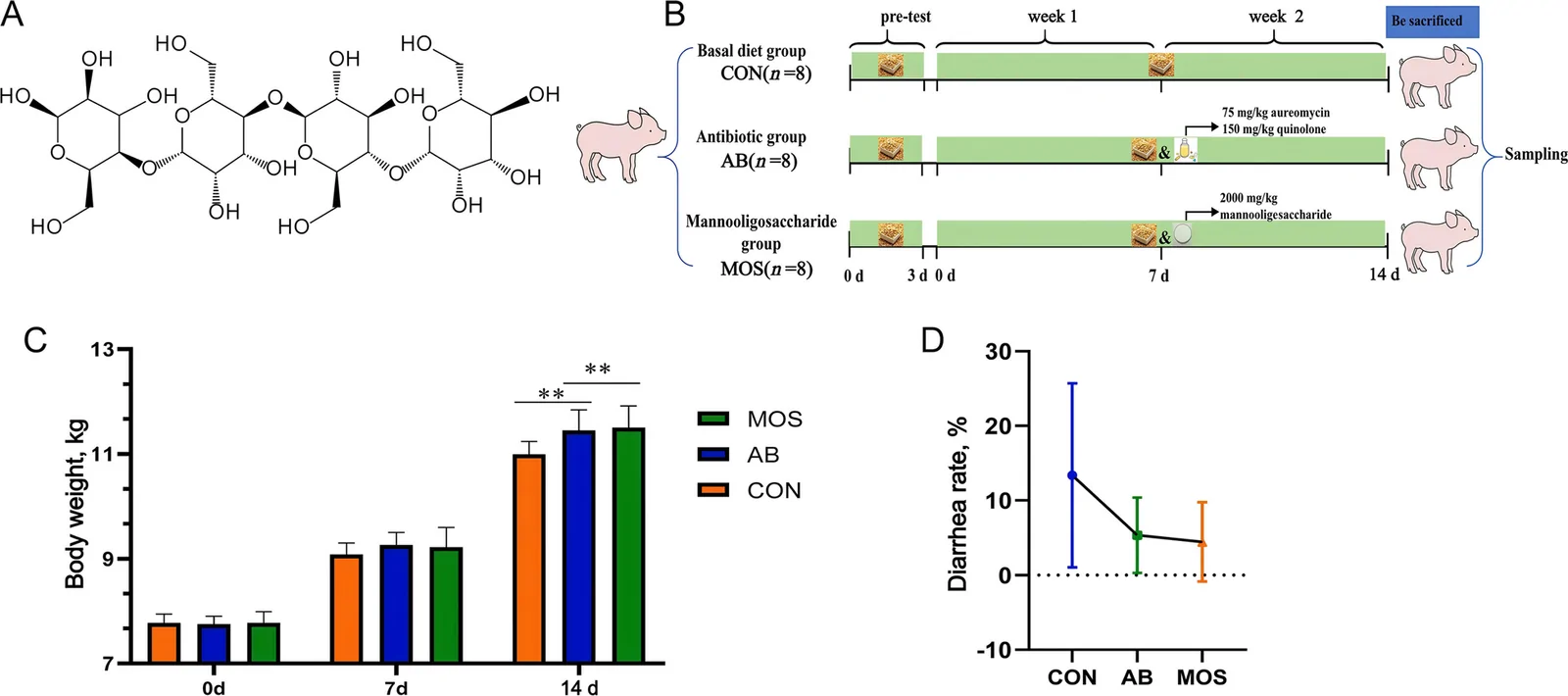
Effects of mannooligosaccharides on growth performance of weaned piglets. **A** Chemical structure formula of mannooligosaccharide. **B** Scheme of the experimental design. **C** Changes of body weight. **D** Diarrhea rate. The data are presented as the mean ± SEM (*n* = 8). CON: corn-soybean based diet; AB: CON + 75 mg/kg aureomycin + 150 mg/kg quinolone; MOS: CON + 2,000 mg/kg MOS. ** indicates *P* < 0.01

By the end of the trial, final BW in the MOS group was notably higher than that in the CON group (*P* = 0.05), with no significant difference between the MOS and AB groups (Fig. 1C). In the initial 7-day period (d 0–7), average daily gain (ADG) was significantly higher in both the AB and MOS groups than in the CON group (*P* = 0.02). In contrast, the average daily feed intake (ADFI) and the gain-to-feed ratio (G:F) did not differ significantly among groups (Table 1). From d 7 to 14, ADG and G:F significantly increased in the AB and MOS groups compared with the CON group, whereas ADFI remained unchanged. No significant difference was observed between the MOS and AB groups. ADFI in the CON group showed no significant change throughout the trial. Notably, the MOS group showed a significantly lower diarrhea rate than the CON group (*P* = 0.05), with no significant difference compared with the AB group (Fig. 1D).

**Table 1:** Effects of mannooligosaccharides on growth performance of weaned piglets

Items	Treatments			SEM	*P* value
	CON	AB	MOS		
0 d body weight, kg	7.78	7.75	7.78	0.07	0.96
7 d body weight, kg	9.08	9.26	9.23	0.11	0.51
14 d body weight, kg	11.00^b^	11.45^a^	11.51^a^	0.15	0.05
Days 0–7					
ADG, g/d	186.96^b^	215.89^a^	207.32^a^	6.20	0.02
ADFI, g/d	335.00	365.36	365.71	15.17	0.29
G:F	0.57	0.60	0.57	0.03	0.79
Days 7–14					
ADG, g/d	273.39^b^	312.86^a^	325.54^a^	7.72	< 0.01
ADFI, g/d	643.43	618.82	623.43	17.32	0.58
G:F	0.43	0.51	0.52	0.02	< 0.01
Days 0–14					
ADG, g/d	230.18^b^	264.38^a^	266.43^a^	6.05	< 0.01
ADFI, g/d	489.22	492.09	494.57	9.57	0.92
G:F	0.47^a^	0.54^b^	0.54^b^	0.01	< 0.01

*SEM* Standard error of the means (*n* = 8), *ADG* Average daily gain, *ADFI* Average daily feed intake, *G:F* Gain:feed

CON: corn-soybean based diet; AB: CON + 75 mg/kg aureomycin + 150 mg/kg quinolone; MOS: CON + 2,000 mg/kg MOS

^a,b^Different superscripts within a row indicate significant differences (*P* < 0.05)

### MOS preserves intestinal barrier integrity in weaned piglets

**Fig. 2:**
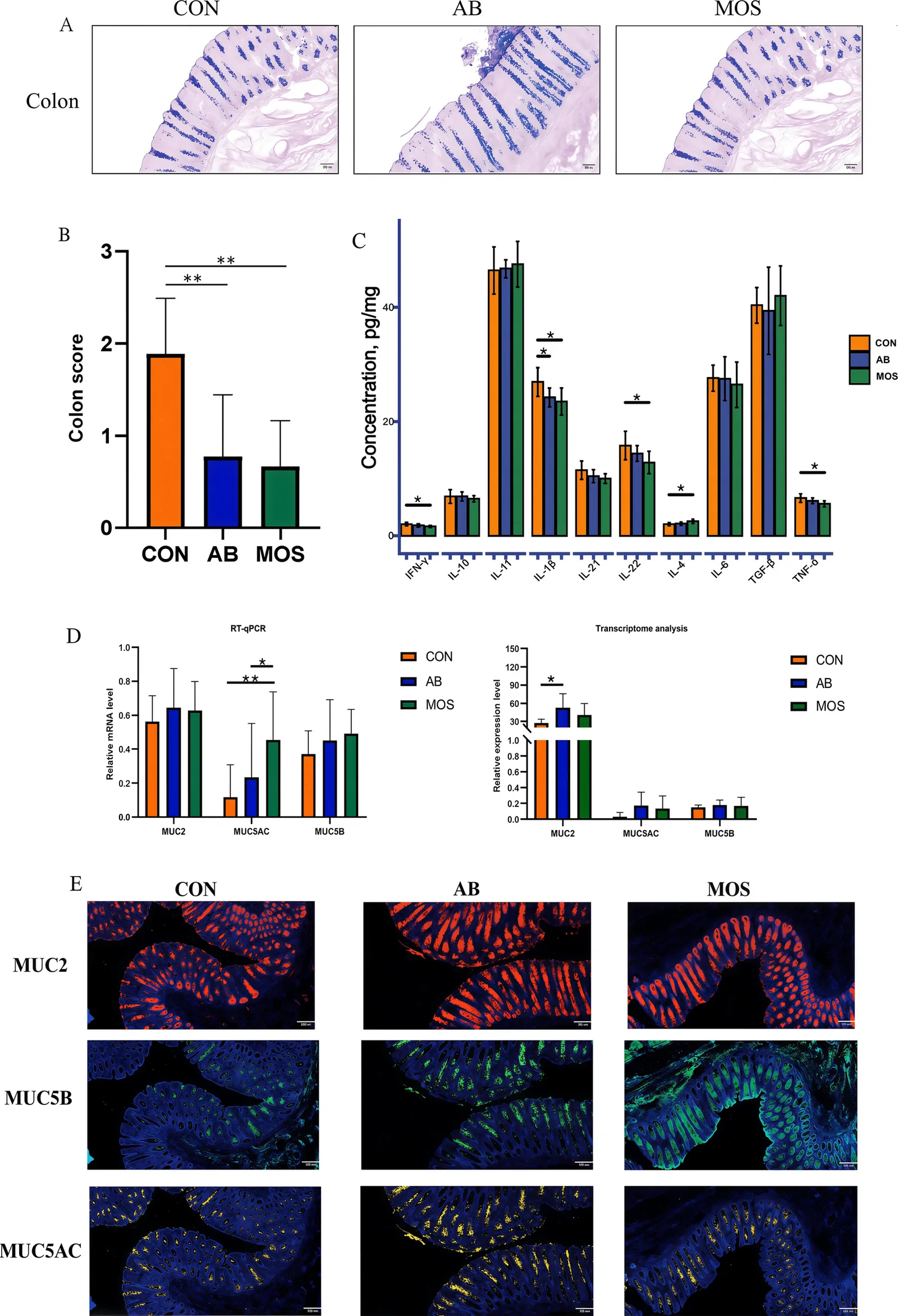
Effects of mannooligosaccharides on intestinal mucosal barrier of weaned piglets. **A** Representative images of AB-PAS-stained colonic sections. Scale bar = 100 μm. **B** The number of goblet cells in the intestine of Histopathological colon score. **C** Content of inflammatory factors in colon tissue. **D** Expression of colonic mucus genes. The data are presented as the mean ± SEM (*n* = 8). **E** By colon section representative immunofluorescence stain, and compare the piglets intestinal mucin gene expression and distribution, such as *MUC2*, *MUC5AC* and *MUC5B*. CON: corn-soybean based diet; AB: CON + 75 mg/kg aureomycin + 150 mg/kg quinolone; MOS: CON + 2,000 mg/kg MOS. ns indicates *P* > 0.05; * indicates 0.05 < *P* < 0.01; ** indicates *P* < 0.01

As shown in Fig. 2A, AB-PAS staining indicated that the CON group showed colonic crypt invagination compared to the MOS and AB groups. Further supporting this finding, histopathological score analysis of colon sections confirmed the reparative effect of MOS on mucosal damage caused by weaning stress (Fig. 2B). Furthermore, examination of pro-inflammatory factors in colon tissue, including IL-22, IFN-γ, IL-1β, and TNF-α, showed that their levels were decreased in the MOS group. In contrast, the level of the anti-inflammatory factor IL-4 was increased and approached that observed in the antibiotic-treated group, indicating that MOS mitigated intestinal inflammation (Fig. 2C). Consistent with these results, RT-qPCR and transcriptomic sequencing showed increased expression levels of mucin-related genes, including* MUC2*, *MUC5AC*, and *MUC5B*, in the colonic mucosa (Fig. 2D). Immunofluorescence analysis of colon tissues confirmed significantly higher expression levels of MUC2, MUC5AC, and MUC5B in the MOS group than in the CON group (Fig. 2E).

### MOS modulates the gut microbiota of weaned piglets

**Fig. 3:**
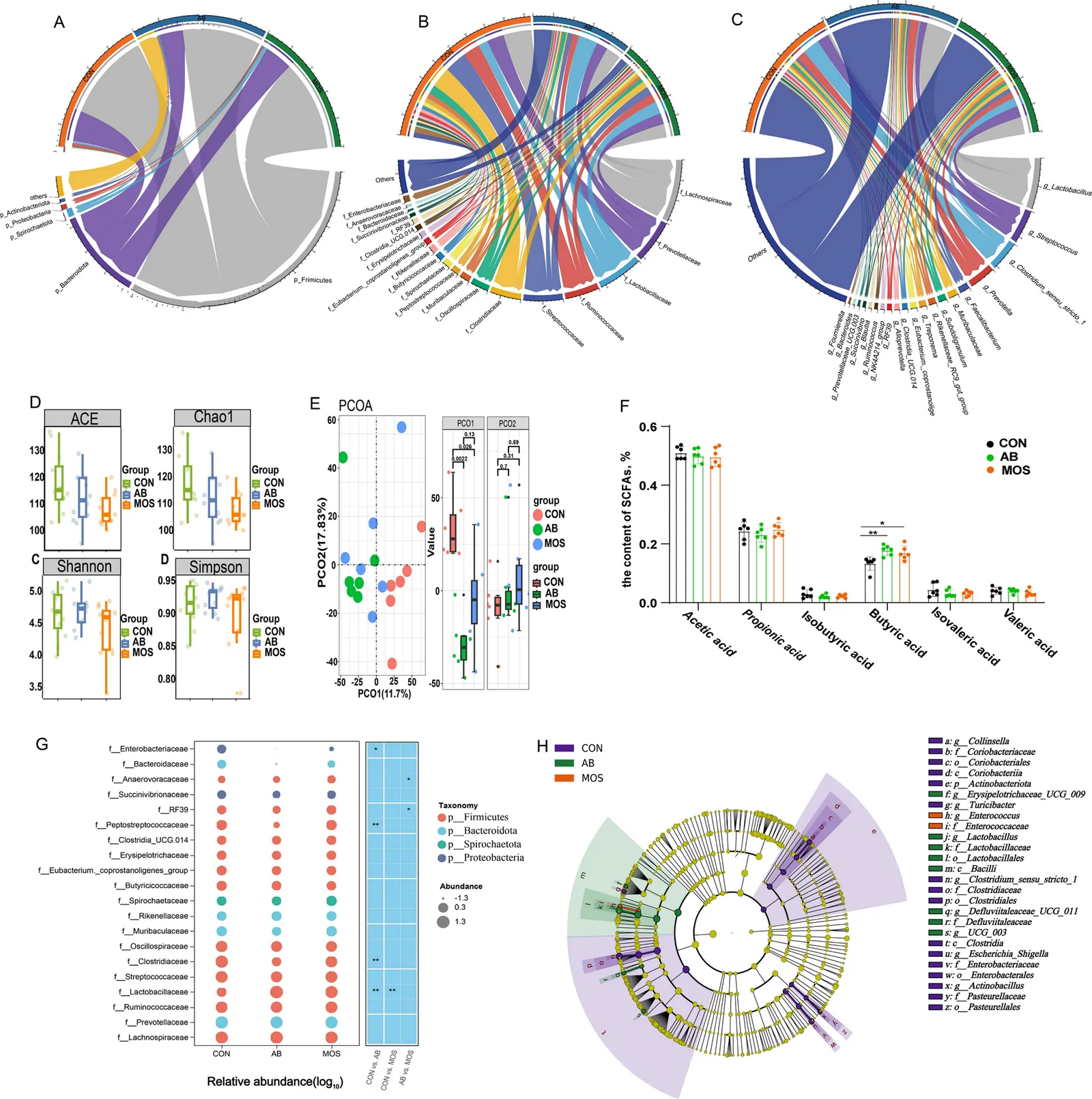
Mannooligosaccharides regulate the composition of intestinal microbes and metabolites of weaned piglets. **A–C **The composition of colonic luminal microbiota at phylum, family, and genus level. **D** α-Diversity of colonic luminal microbiota. **E** β-Diversity of colonic luminal microbiota. **F** Short-chain fatty acid content in colonic luminal. **G** Heatmap analysis of the composition of colonic luminal microbiota at the family level. **H** LEfSe analysis of colonic luminal microbiota. CON: corn-soybean based diet; AB: CON + 75 mg/kg aureomycin + 150 mg/kg quinolone; MOS: CON + 2,000 mg/kg MOS. * indicates *P* < 0.05; ** indicates *P* < 0.01

When MOS was incorporated into the diets of weaned piglets, significant alterations in gut microbiota composition were observed. At the phylum level, the most abundant taxa in colonic contents were Firmicutes, Bacteroidota, Spirochaetota, Proteobacteria, and Actinobacteriota, in that order (Fig. 3A). Notably, MOS supplementation increased the relative abundance of Bacteroidota and decreased that of Proteobacteria compared with the CON group, although these changes did not reach statistical significance. The dominant families in colonic contents were Lachnospiraceae, Prevotellaceae, Lactobacillaceae, Ruminococcaceae and Streptococcaceae (Fig. 3B). Meanwhile, at the genus level, the main bacteria in colonic contents were *Lactobacillus*, *Streptococcus*, *Clostridium_sensu_stricto_1*, *Prevotella*, and *Faecalibacterium* (Fig. 3C). In addition, analysis of microbial diversity in piglet colonic contents revealed no significant differences in α-diversity, although the Chao1 index showed a decreasing trend (Fig. 3D). In the AB group, antibiotic treatment induced notable changes in gut microbiota composition. Compared with the CON group, the AB group showed a decreasing trend in α-diversity (Chao1 index, *P* = 0.08) and a distinct clustering pattern in β-diversity analysis. Interestingly, β-diversity analysis of piglet colonic contents revealed a distinct separation trend. For the colonic microbiota, the MOS group significantly differed from the CON group at the PC1 level (*P* = 0.03), with no significant difference compared with the AB group. These findings indicate that dietary MOS supplementation induces shifts in the microbial composition of piglets (Fig. 3E).

Family-level heatmap analysis revealed clear differences in the microbial composition of piglet colonic contents. Lactobacillaceae was the predominant family in the colonic contents, as shown in Fig. 3G. To identify differentially abundant taxa following MOS supplementation, LEfSe analysis was performed (LDA score > 3.0, *P* < 0.05). As shown in Fig. 3H and Table S3, the MOS group exhibited a significant enrichment of *Lactobacillus* (LDA = 4.92, *P* = 0.00395) and other members of the Lactobacillales order (LDA = 4.91, *P* = 0.01041). In contrast, the CON group was characterized by a higher abundance of potentially pathogenic taxa, including *Escherichia_Shigella* (LDA = 3.87, *P* = 0.02497), *Actinobacillus* (LDA = 3.59, *P* = 0.02223), and *Turicibacter* (LDA = 3.81, *P* = 0.01448). The MOS group showed reduced relative abundance of deleterious bacteria, such as *Collinsella*, *Escherichia_Shigella*, and *Actinobacillus*, together with increased abundance of *Lactobacillus* (Table S3). Concurrently, the concentrations of SCFAs, pivotal metabolites of the colonic microbiota, were quantified in piglet colonic contents. Among the measured SCFAs, including acetate, propionate, and butyrate. Butyrate concentration was notably increased in both the MOS and AB groups compared with the CON group, whereas other fatty acids showed no statistically significant changes (Fig. 3F). In summary, supplementing MOS to piglet feed regulated intestinal microbiota composition and maintained gut microbial equilibrium.

### MOS regulates the expression of intestinal mucosal glycosylation genes in weaned piglets

**Fig. 4:**
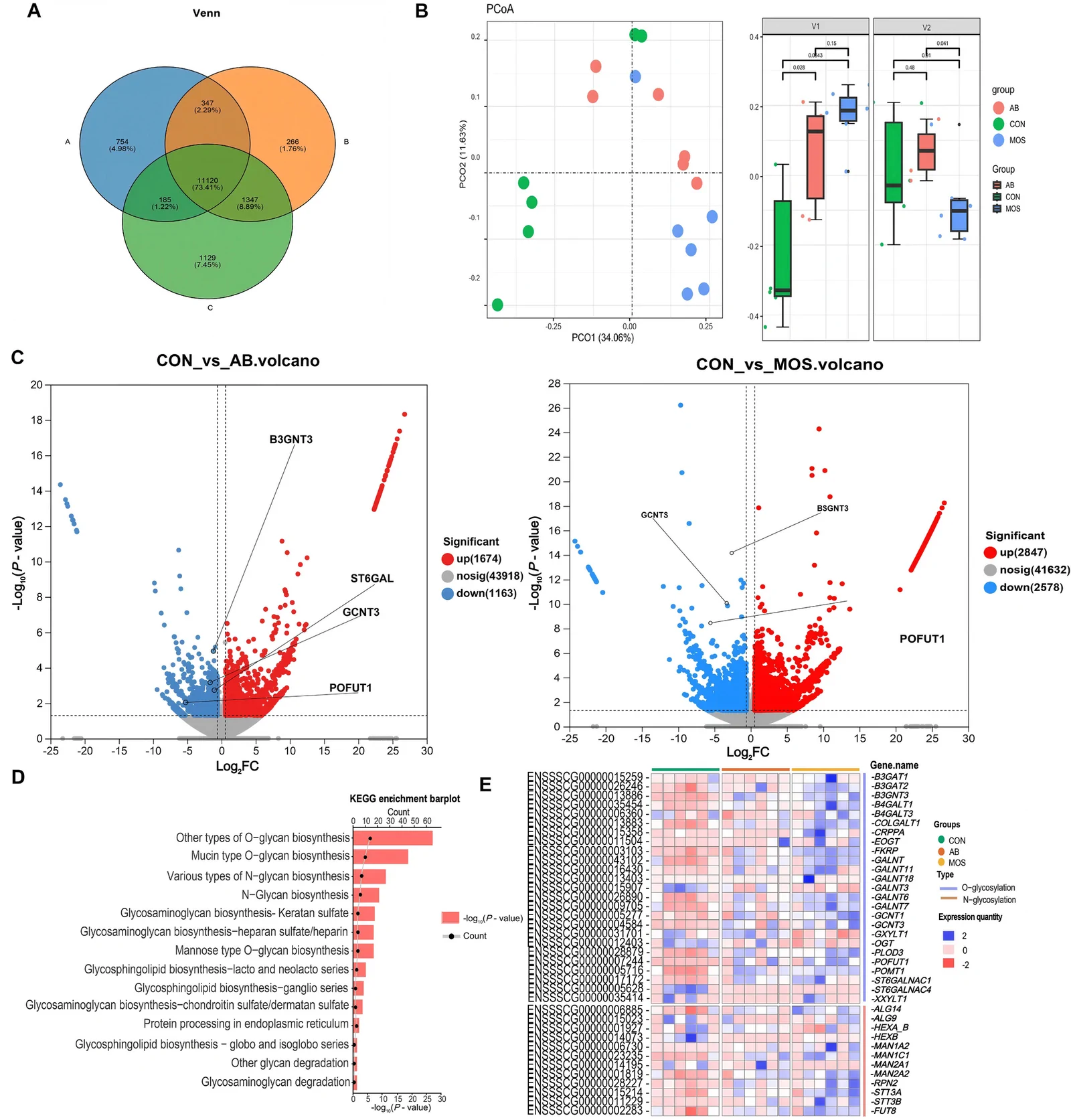
Mannooligosaccharides change the expression of intestinal glycosylation-related genes in weaned piglets. Transcriptome analysis of colon mucosa of piglets. **A** Venn diagram. **B** Principal component analysis studies inter-sample correlation. **C** Volcano plot. **D** KEGG enrichment analysis. **E** Heatmap of differentially expressed genes. CON: corn-soybean based diet; AB: CON + 75 mg/kg aureomycin + 150 mg/kg quinolone; MOS: CON + 2,000 mg/kg MOS

Eight colonic mucosal samples from the MOS group, along with 12 colonic mucosal samples from the AB group and another 12 from the CON group, underwent high-throughput transcriptome sequencing. A total of 279.29 Gb of clean data were obtained, and the clean data of all samples exceeded 6.16 Gb, with Q30 base percentages greater than 91.37%. Six samples were randomly selected from each group for follow-up analysis. As shown in Fig. 4A, unique gene counts in the MOS, AB, and CON groups were 1,129 (7.45%), 266 (1.76%), and 754 (4.98%), respectively. The number of genes shared across all three groups was 11,120, accounting for 73.41% of the total. Notably, the MOS and CON groups showed a limited overlap, sharing only 185 genes, equivalent to 1.22%. Principal component analysis was also performed to assess inter-sample correlations based on individual gene expression levels. The samples from the three groups showed a separation trend, and the MOS and AB groups were significantly separated from the CON group at the PC1 level, with low correlation (Fig. 4B).

Differential expression analysis identified 2,578 and 1,163 upregulated transcripts in the MOS and AB groups, respectively, compared with the CON group. Meanwhile, 2,847 and 1,674 transcripts were downregulated expression. In addition, 41,632 and 43,918 transcripts showed no significant expression changes. KEGG enrichment analysis indicated that MOS affected pathways related to glycan synthesis, particularly mucin-type O-glycan biosynthesis, various types of N-glycan biosynthesis, and mannose-type O-glycan biosynthesis, supporting our earlier findings from GO functional annotation analysis (Fig. 4D). To further validate the impact of MOS on glycosylation-related genes, we generated heatmaps for DEGs involved in O-glycan and N-glycan glycosylation. These heatmaps demonstrated significant upregulation of O-glycosylation-related genes in both the MOS and AB groups. Notably, B3GNT3, GCNT3, and POFUT1 were consistent with the volcano plot results (Fig. 4E).

### MOS improves the O-glycosylation profile of the colonic mucus layer in weaned piglets

**Fig. 5:**
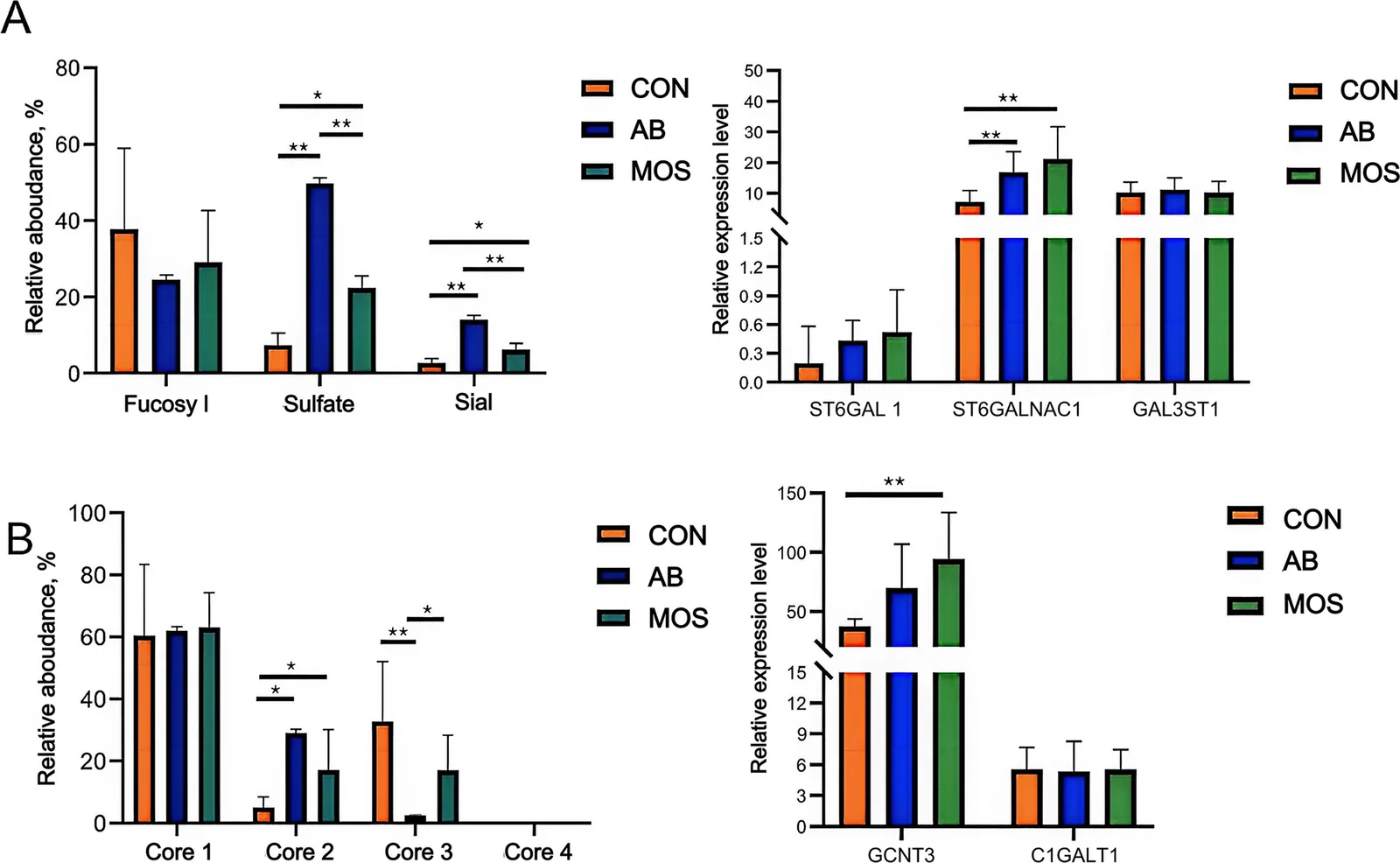
Effects of mannooligosaccharides on the glycosylation of mucin O-glycans in piglets. **A** The expression of O-glycation modified structure types and glycosyltransferases in the colon of piglets. **B** The expression of O-glycation modified core structures and glycosyltransferases in the colon of piglets. * indicates *P* < 0.05; ** indicates *P* < 0.01

**Fig. 6:**
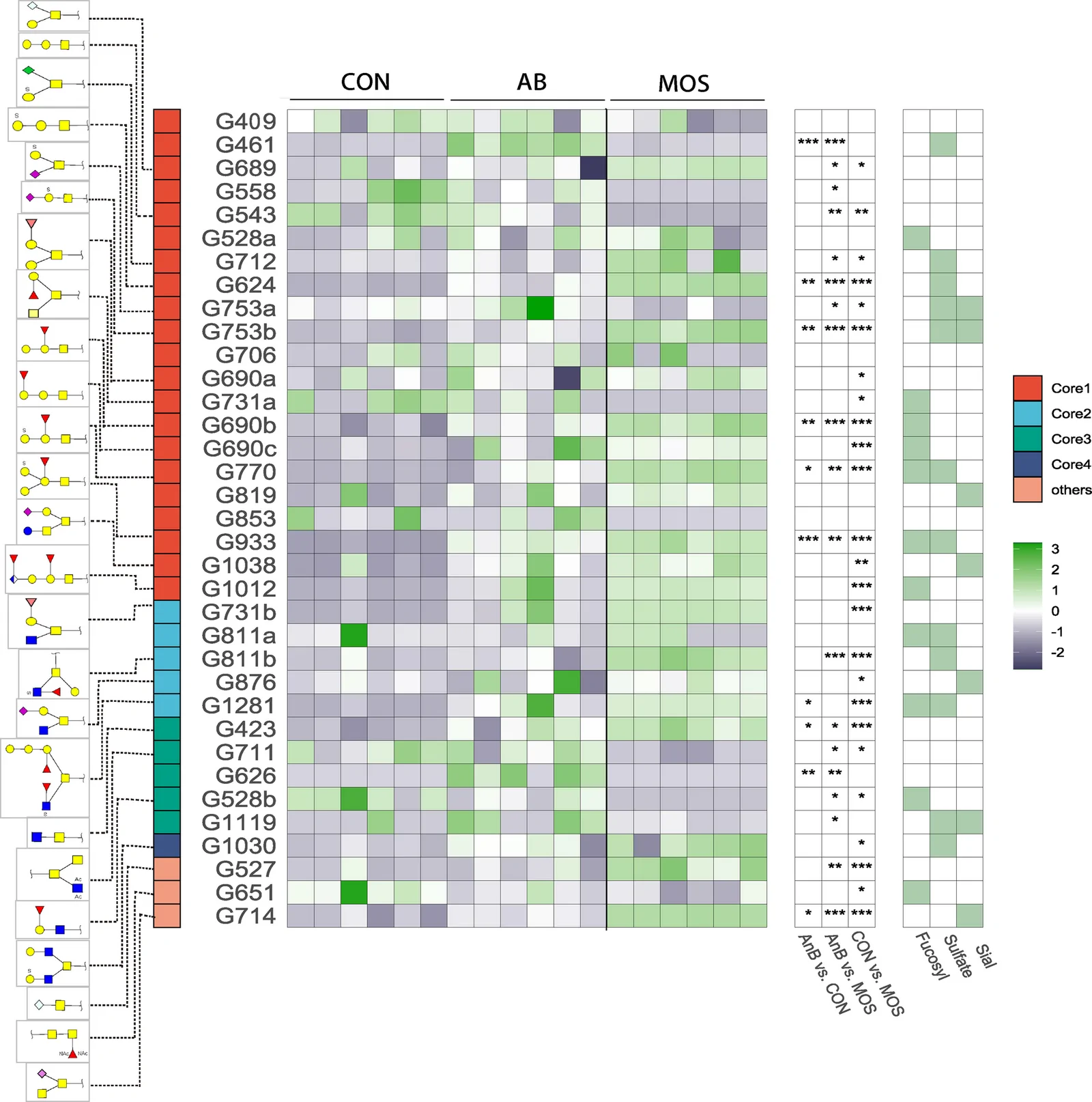
Mannooligosaccharides change the O-glycosylation spectrum and the long and short chain ratio of the colon mucous layer of piglets. Heatmap illustrating the altered relative abundance of mucin O-glycans of colonic mucosa in piglets. CON: corn-soybean based diet; AB: CON + 75 mg/kg aureomycin + 150 mg/kg quinolone; MOS: CON + 2,000 mg/kg MOS. * indicates *P* < 0.05; ** indicates *P* < 0.01; *** indicates *P* < 0.001

Analysis of the O-glycan composition in piglet colonic mucins identified 35 distinct O-glycans with various core types, predominantly Core 1 and Core 2 structures, with fewer Core 3, Core 4, and other structures. Subsequent analysis revealed pronounced upregulation of Core 2 structures in the MOS group compared with the CON group. This enhancement was paralleled by a significant increase in the expression of related genes, such as* GCNT3* (Fig. 5B). MOS can affect O-glycan glycosylation modification and alter the stability of carbohydrate structures. Overall, 10 types of O-glycans were identified in the MOS group, mainly Core 2 structures, including 4 fucosylated, 6 sulfated, and 2 sialylated structures. Meanwhile, the MOS group showed pronounced increases in both sialylated and sulfated mucin expression levels compared with the CON group. Subsequent transcriptomic analysis revealed an upward trend in the expression levels of glycosyltransferase genes involved in glycosylation modifications, particularly *ST6GAL1*. A significant increase was also observed in ST6GALNAC1 expression (Fig. 5A). G1281, G933, G770, G753b, G624, and G690b are long-chain O-glycans containing 3–7 monosaccharide units. In contrast, G543 and G461 represent short-chain O-glycans. Relative abundance analysis indicated an increase in long-chain O-glycans and a decrease in short-chain O-glycans in the colons of MOS-fed piglets. Moreover, the relative abundance of O-glycan core structures and glycosylation modification structures was compared among the three treatment groups. The results revealed significant increases in the proportions of Core 2 and Core 3 O-glycans in both the MOS and AB groups. In addition, O-glycan sulfation and sialylation were notably increased under antibiotic and MOS treatment (Fig. 6).

### MOS regulates the relationship between mucin O-glycans and the piglet microbiota

**Fig. 7:**
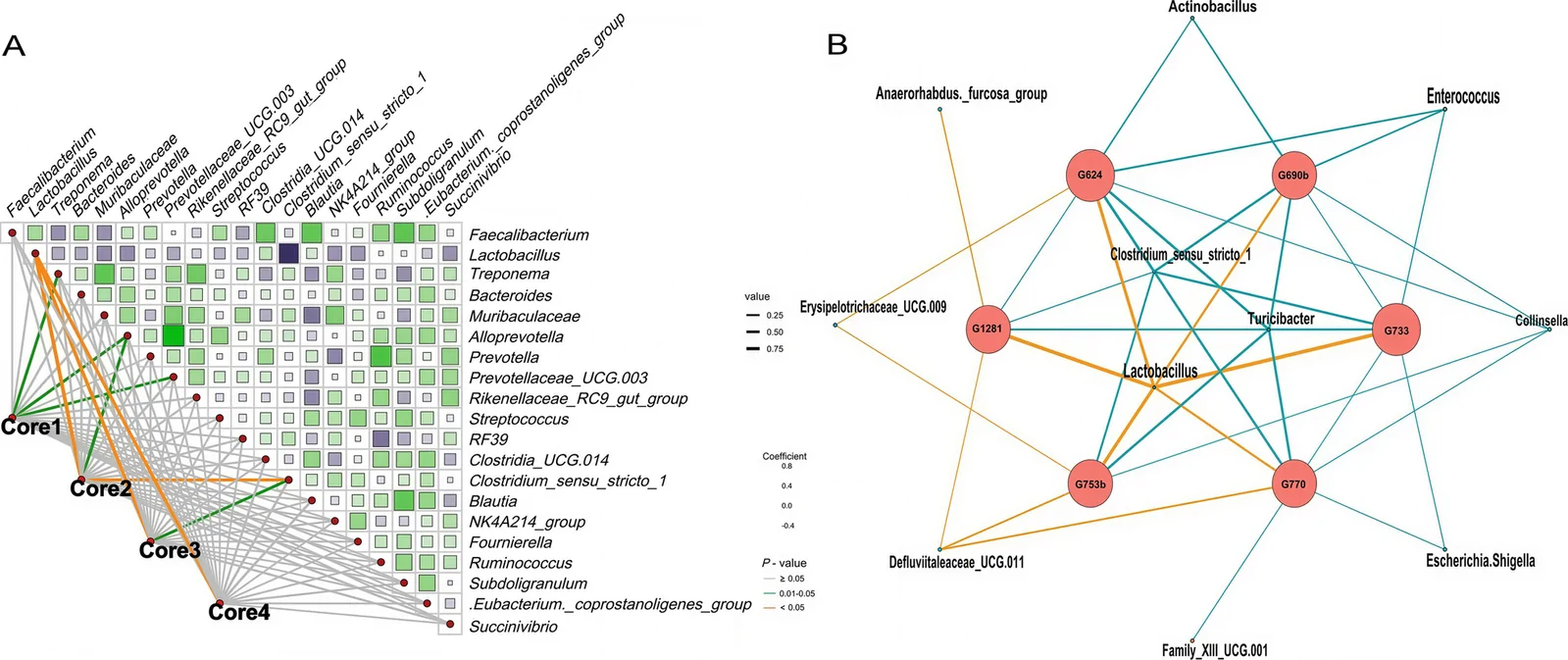
Mannooligosaccharides regulate the relationship between mucin O-glycans and the microbiota in piglets. **A** O-glycans are related to each taxon by partial Mantel tests. **B** Network analysis of the correlations between the gut microbiota and 6 characteristic O-glycan biomarkers

To reveal the relationship between MOS-induced changes in O-glycan composition and intestinal microbes, we performed correlation network analysis of differential colonic microbes and mucin O-glycan structures. The results showed that *Lactobacillus* was closely associated with O-glycans, including Core 2, Core 3, and Core 4 structures. *Treponema*, *Alloprevotella*, and *Prevotellaceae_UCG.003* showed strong correlations with Core 1, while Core 3 and Core 4 were also strongly associated with *Clostridium_sensu_stricto_1* (Fig. 7A). We also conducted correlation network analysis between 6 differential O-glycans, including G1281, G933, and G753b, with different colonic microbes. Increases in G1281, G933, G753b, G770, G624, and G690b were positively correlated with *Lactobacillus*. Although these O-glycans were negatively correlated with *Clostridium_sensu_stricto_1*, the abundances of G770 and G933 showed an inverse relationship with *Escherichia coli*. Furthermore, the abundances of G753b, G770, G624, and G690b were negatively correlated with *Collinsella*, *Enterococcus*, and various other strains (Fig. 7B). These findings were consistent with the LEfSe analysis, which showed that the relative abundance of *Lactobacillus* increased in the intestinal tracts of MOS-fed piglets, whereas the relative abundances of *Collinsella*, *Escherichia coli*, and *Enterococcus* decreased (Fig. 3H). These findings indicate that dietary MOS supplementation can alter the microbial composition of piglets, increase the proportion of long-chain O-glycans, and improve O-glycan structure. This regulation contributed to the maintenance of intestinal microflora homeostasis, thereby creating a favorable environment for mucin O-glycan–microbiota interactions and ultimately promoting animal health.

## Discussion

Weaning stress-induced intestinal dysfunction is a prevalent challenge in modern swine production, resulting in reduced growth performance, increased morbidity, and substantial economic losses to the pork industry. The normal intestinal symbiotic system possesses intrinsic resistance mechanisms against pathogenic invasion; however, antibiotic exposure can weaken or even abolish this protective capacity. Prolonged dietary antibiotic use can disrupt the composition of gastrointestinal microorganisms, compromising intestinal barrier function and increasing the risk of enteric disorders [24–26]. Given the growing concerns over antimicrobial resistance and the global ban on antibiotic growth promoters, there is an urgent need to identify effective alternatives to in-feed antibiotics [27]. In recent years, functional carbohydrates, particularly MOS, have emerged as promising feed additives for promoting intestinal health in weaned piglets [28].

Our study supports existing evidence that MOS can enhance growth performance, feed conversion, and intestinal health in weaned piglets. Compared with the CON group, the supplementation of 0.2% MOS in the diet significantly increased final BW, ADG, and feed efficiency in weaned piglets. In addition, the diarrhea rate was significantly reduced and did not differ from that in the AB group. These findings underscore the capacity of MOS to improve piglet growth, enhance feed utilization, and reduce diarrhea incidence, thereby maintaining intestinal health. Similarly, Che et al. [29] demonstrated that dietary MOS reduced the porcine reproductive and respiratory syndrome virus (PRRSV) induced decrease in G:F in infected piglets. Additional studies have confirmed the positive effects of MOS on final BW, average daily gain, and feed-to-gain ratio in weaned piglets, as well as its ability to reduce diarrhea rates [30–32]. These consistent results support the reliability of our findings.

As a functional oligosaccharide, MOS can be fermented by specific intestinal bacteria, leading to SCFA production and an acidic environment conducive to intestinal epithelial cell proliferation. This, in turn, benefits intestinal morphology and mucosal integrity [33–35]. Yu et al. [36] reported that supplementing MOS to the diets of weaned piglets challenged with *Escherichia coli* significantly improved the duodenal and ileal villus and the villus height-to-crypt depth ratio, thereby effectively improving nutrient utilization in weaned piglets. Similarly, studies in broilers have shown that MOS can reduce mucosal damage by increasing jejunal villus height and the villus height-to-crypt depth ratio, thereby supporting intestinal mucosal barrier integrity [37]. Our study is consistent with these previous findings, demonstrating that MOS can improve intestinal crypt and villus development in weaned piglets and promote the development of goblet cells and the production of secretions such as MUC2 and MUC5AC. These effects help maintain intestinal mucosal barrier integrity and strengthen its defensive function. In conclusion, dietary MOS supplementation has important reference value for maintaining normal intestinal morphology and provides a new approach for treating mucosal injury in weaned piglets.

The intestinal mucosal barrier serves as the body's first line of defense against foreign invaders and plays a crucial role in intestinal health [38]. As an important molecular component of the mucus barrier, mucin plays a key role in this process. O-glycans, which extend from the mucin core and constitute a major component of mucin, are closely associated with the external environment and mucus barrier function [39]. The interaction among the intestinal mucus layer, microbiota, and host epithelium collectively establishes a pattern of symbiotic homeostasis, which is highly important for overall health [40]. In recent years, studies have clarified the nature and extent of the influence of mucin O-glycans on intestinal homeostasis, demonstrating their role in preventing intestinal diseases [41].

Moreover, intestinal microorganisms, in turn, affect the formation of the intestinal mucosa and O-glycans to maintain intestinal environmental homeostasis. Metabolites produced by intestinal bacteria can directly enhance the mucosal barrier function of intestinal epithelial cells. Among these metabolites, butyrate, a SCFA produced by intestinal bacteria, can stimulate mucin secretion from goblet cells [42]. Importantly, intestinal microorganisms can also acquire, release, and consume O-glycans as carbon sources. For example, *Bifidobacterium myxophilus*, *Bifidobacterium,* and *Bacteroides* are prominent O-glycan-utilizing bacteria that influence and regulate host inflammatory responses [43]. It has been reported that carbohydrates can affect microbial species composition, but their regulatory effects on O-glycans have rarely been studied [44]. In this study, we identified the role of MOS in maintaining intestinal microflora homeostasis. After MOS treatment, the relative abundance of *Escherichia_Shigella* in the colon decreased compared with the CON group, and butyrate concentration increased in the MOS group. In addition, our data showed that AB treatment also modulated the gut microbiota, including a reduction in α-diversity and enrichment of *Lactobacillus*. Although the microbiota changes induced by MOS and AB treatments shared some similarities, the antibiotic group exhibited more pronounced suppression of diversity compared with the MOS group, which may partially explain the differential effects of these two interventions on intestinal homeostasis. These results are consistent with previous studies, indicating that dietary MOS can effectively regulate the intestinal microbial structure and metabolite production in piglets.

Mucin plays an important role in mucosal immunity, and O-glycans, which account for up to 80% of mucin, act as a protective shield against bacterial protease degradation and safeguard intestinal epithelial cells from microbiota-dependent colitis. Moreover, diverse glycosylation modifications enable O-glycans to play an important role in maintaining intestinal homeostasis [7, 45], and intestinal mucin glycosylation is regulated by environmental signals from the microbiome and diet [46, 47]. MOS can maintain normal intestinal mucosal development and regulate intestinal microbial composition in piglets, while mucosal injury and chronic intestinal inflammation are closely related to epithelial cell glycosylation [48]. PGC-LC–MS/MS was used to detect changes in O-glycan chains and explore the regulatory mechanism of MOS on mucin O-glycosylation. O-glycans include four main core structures (Core 1–Core 4), and their carbohydrate chains vary in length. Mucin O-glycans are modified to form sialylated, fucosylated, and sulfated O-glycans, which are closely related to the pathogenesis of mucosal injury [47, 49, 50]. The glycosylation of O-glycans mainly includes fucosylation, sulfation, and sialylation, and most O-glycans are sialylated and sulfated in the healthy human colon. Sialic acid, in particular, plays critical roles in cell adhesion and host–pathogen signaling. Notably, O-glycan sulfation is reduced in the gut of patients with IBD. Increasing sulfation modifications can restore mucin glycosylation to normal levels and alleviate intestinal inflammation [50]. Our PGC-LC–MS/MS analysis of colon mucosa revealed significant increases in sulfation and sialylation modifications in the MOS group, accompanied by a tendency toward elongated sugar chains, most notably in the long-chain O-glycans G1281, G933, G753b, G770, G624, and G690b. These results showed that intestinal mucin O-glycans in piglets were markedly altered after MOS supplementation. The results above also indicate that MOS plays a beneficial role in the intestinal microbiota structure of piglets. Together, these findings confirm that MOS is involved in the interaction between O-glycans and microorganisms. Nevertheless, determining the causal relationship between microorganisms and MOS-regulated O-glycan modification remains a complex challenge that requires further research.

Intestinal glycosylation plays an important role in maintaining homeostasis between the intestinal epithelium and microorganisms [51]. Most mucin core glycans are O-glycans. Functional carbohydrates, as specific nutrients in the body, serve as immunomodulators in the intestinal mucosa. However, current research lacks systematic screening of functional genes that regulate O-glycan synthesis in response to functional carbohydrates [52]. In this study, transcriptomic analysis showed that the expression levels of genes related to O-glycan synthesis, such as *GCNT3* and *B3GNT3*, were increased in the MOS group, and the expression levels of mucins such as *MUC2* and *MUC5AC* also tended to increase. Among the upregulated transcripts, *GCNT3*, *B3GNT3*, and *POFUT1* were highlighted because these genes are pivotal in mucin glycosylation (Fig. 4C). These three genes are essential mucin glycosylation molecules. Among them, *GCNT3* encodes C2/4GnT glycosyltransferase, which catalyzes the formation of Core 2, Core 3, and Core 4 O-glycans [23]. RNA-Seq was used for GO functional annotation analysis, which mainly included three aspects: biological process, cellular component, and molecular function. Metabolic and cellular processes were the main changes among biological processes, suggesting that MOS is closely related to cell growth, reproduction, apoptosis, and subsequent growth and development, and plays an important role in maintaining environmental balance. The main changes in cellular components involved cell part, membrane, organelle part, membrane part, and organelle, suggesting that dietary MOS can change the biological composition of cells and affect piglet organelles. At the molecular function level, the predominant changes were observed in binding and catalytic activities. This observation underscores the close relationship between MOS and protein functional attributes, as well as the catalytic capabilities of associated enzymes (Table 1).

KEGG enrichment analysis highlighted the mucin-type O-glycan biosynthesis pathway as the predominant pathway in the mannooligosaccharide group. Studies have shown that GCNT3 expression is increased in diseased mice and human cancer, and inhibiting its synthesis disrupts mucin formation, thereby affecting the immune function of the mucosal barrier. Changes in B3GNT3 expression have been associated with T cell-mediated antitumor immunity. Earlier research has also found that the activity of mucin glycan synthesis genes, including *GCNT3*, plays a significant role in physiological processes such as inflammation and immune responses [53, 54]. Our findings align with previous studies, highlighting the capacity of mannooligosaccharides to regulate the expression of genes involved in glycan synthesis and thereby modulate mucosal immunity. These data provide valuable support for subsequent practical applications in production and research.

## Conclusions

In conclusion, our study demonstrates that dietary MOS supplementation can enhance piglet growth, maintain the host-O-glycan-intestinal microbe axis, and regulate intestinal microbiota disturbances and barrier dysfunction after weaning. MOS helps piglets overcome weaning stress and provides valuable insights into the potential of new prebiotics for treating intestinal inflammation and other piglet diseases. Furthermore, this research paves the way for deeper investigations into the complex interaction between mucin O-glycans and the intestinal microbiota in health and disease, shedding light on the underlying mechanisms through which MOS promotes intestinal health.

## Supplementary Information

Additional file 1: Table S1. Composition and nutrient levels of the experimental diet. Table S2. Primer sequence information. Table S3. Taxa altered in the colon of piglets.

## Data Availability

The data used or analysed during the current study are available from the corresponding author on reasonable request.

## Abbreviations

AB-PAS: Alcian blue-periodic acid-Schiff
ADFI: Average daily feed intake
ADG: Average daily gain
B3GNT3: Beta-1,3-N-acetylglucosaminyltransferase 3
BW: Body weight
DEPC: Diethyl pyrocarbonate
DTT: Dithiothreitol
G:F: Gain-to-feed ratio
HPGC: High-performance gas chromatography
IAA: Iodoacetamide
IBD: Inflammatory bowel disease
IL-1β: Interleukin-1β
IL-22: Interleukin-22
IL-4: Interleukin-4
IFN-γ: Interferon-gamma
LC–MS/MS: Liquid chromatography-tandem mass spectrometry
MOS: Mannooligosaccharides
MUC: Mucin
PRRSV: Porcine reproductive and respiratory syndrome virus
SCFA: Short-chain fatty acid
TNF-α: Tumor necrosis factor-alpha

## References

[1] Wehkamp J, Götz M, Herrlinger K, Steurer W, Stange EF. Inflammatory bowel disease: Crohn’s disease and ulcerative colitis. Dtsch Arztebl Int. 2016;113(5):72. 10.3238/arztebl.2016.0072. 26900160 10.3238/arztebl.2016.0072 PMC4782273

[2] Boltin D, Perets TT, Vilkin A, Niv Y. Mucin function in inflammatory bowel disease. J Clin Gastroenterol. 2013;47(2):106–11. 10.1097/MCG.0b013e3182688e73. 23164684 10.1097/MCG.0b013e3182688e73

[3] Arike L, Hansson GC. The densely O-glycosylated MUC2 mucin protects the intestine and provides food for the commensal bacteria. J Mol Biol. 2016;428(16):3221–9. 10.1016/j.jmb.2016.02.010. 26880333 10.1016/j.jmb.2016.02.010 PMC4982847

[4] Bergstrom K, Shan XD, Casero D, Batushansky A, Lagishetty V, Jacobs JP, et al. Proximal colon-derived O-glycosylated mucus encapsulates and modulates the microbiota. Science. 2020;370(6515):467–72. 10.1126/science.aay7367 10.1126/science.aay7367 PMC8132455 33093110

[5] Lee IK, Kye YC, Kim G, Kim HW, Gu MJ, Umboh J, et al. Stress, nutrition, and intestinal immune responses in pigs — a review. Asian-Australas J Anim Sci. 2016;29(8):1075–82. 10.5713/ajas.16.0118. 27189643 10.5713/ajas.16.0118 PMC4932560

[6] Vaga S, Lee S, Ji B, Andreasson A, Talley NJ, Agréus L, et al. Compositional and functional differences of the mucosal microbiota along the intestine of healthy individuals. Sci Rep. 2020. 10.1038/s41598-020-71939-2. 10.1038/s41598-020-71939-2 PMC7486370 32917913

[7] Johansson MEV, Hansson GC. Immunological aspects of intestinal mucus and mucins. Nat Rev Immunol. 2016;16(10):639–49. 10.1038/nri.2016.88. 27498766 10.1038/nri.2016.88 PMC6435297

[8] Buisine M, Desreumaux P, Leteurtre E, Copin M, Colombel J, Porchet N, et al. Mucin gene expression in intestinal epithelial cells in Crohn’s disease. Gut. 2001;49(4):544–51. 10.1136/gut.49.4.544. 11559653 10.1136/gut.49.4.544 PMC1728475

[9] Dorofeyev AE, Vasilenko IV, Rassokhina OA, Kondratiuk RB. Mucosal barrier in ulcerative colitis and crohn's disease. Gastroent Res Pract. 2013. 10.1155/2013/431231 10.1155/2013/431231 PMC3664489 23737764

[10] Silverberg MS, Satsangi J, Ahmad T, Arnott ID, Bernstein CN, Brant SR, et al. Toward an integrated clinical, molecular and serological classification of inflammatory bowel disease: report of a working party of the 2005 Montreal world congress of gastroenterology. Can J Gastroenterol. 2005;19:5A-36A. 10.1155/2005/269076. 16151544 10.1155/2005/269076

[11] Tannock GW. Building robust assemblages of bacteria in the human gut in early life. Appl Environ Microbiol. 2021. 10.1128/AEM.01449-21. 34469198 10.1128/AEM.01449-21 PMC8552902

[12] Zhang Q, Jin K, Chen B, Liu R, Cheng S, Zhang Y, et al. Overnutrition induced cognitive impairment: insulin resistance, gut-brain axis, and neuroinflammation. Front Neurosci. 2022;16:884579. 10.3389/fnins.2022.884579. 35873818 10.3389/fnins.2022.884579 PMC9298971

[13] Faustino M, Durao J, Pereira CF, Pintado ME, Carvalho AP. Mannans and mannan oligosaccharides (MOS) from *Saccharomyces cerevisiae* – A sustainable source of functional ingredients. Carbohyd Polym. 2021. 10.1016/j.carbpol.2021.118467. 10.1016/j.carbpol.2021.118467 34420726

[14] Liu N, Shen HB, Zhang F, Liu X, Xiao QR, Jiang Q, et al. Applications and prospects of functional oligosaccharides in pig nutrition: a review. Anim Nutr. 2023;13:206–15. 10.1016/j.aninu.2023.02.002. 37388461 10.1016/j.aninu.2023.02.002 PMC10300388

[15] Xue SX, Chen XM, Lu JX, Jin LQ. Protective effect of sulfated polysaccharides on streptozotocin-induced oxidative stress in rats. Carbohyd Polym. 2009;75(3):415–9. 10.1016/j.carbpol.2008.08.003.

[16] Halas V, Nochta I. Mannan oligosaccharides in nursery pig nutrition and their potential mode of action. Animals (Basel). 2012;2(2):261. 10.3390/ani2020261. 26486920 10.3390/ani2020261 PMC4494321

[17] Kiczorowska B, Samolinska W, Al-Yasiry ARM, Kiczorowski P, Winiarska-Mieczan A. The natural feed additives as immunostimulants in monogastric animal nutrition - a review. Ann Anim Sci. 2017;17(3):605–25. 10.1515/aoas-2016-0076.

[18] Zhang Q, Widmer G, Tzipori S. A pig model of the human gastrointestinal tract. Gut Microbes. 2013;4(3):193–200. 10.4161/gmic.23867. 23549377 10.4161/gmic.23867 PMC3669164

[19] National Research Council. Nutrient requirements of swine. 11th revised ed. Washington, DC: The National Academies Press; 2012. 10.17226/13298.

[20] Agazzi A, Perricone V, Zorini FO, Sandrini S, Mariani E, Jiang XR, et al. Dietary mannan oligosaccharides modulate gut inflammatory response and improve duodenal villi height in post-weaning piglets improving feed efficiency. Animals. 2020. 10.3390/ani10081283. 32731342 10.3390/ani10081283 PMC7459834

[21] Huang S, Cui Z, Hao X, Cheng C, Chen J, Wu D, et al. Dietary fibers with low hydration properties exacerbate diarrhea and impair intestinal health and nutrient digestibility in weaned piglets. J Anim Sci Biotechnol. 2022;13:142. 10.1186/s40104-022-00771-7. 36352481 10.1186/s40104-022-00771-7 PMC9644590

[22] Xia B, Zhong RQ, Wu WD, Luo CZ, Meng QS, Gao QT, et al. Mucin O-glycan-microbiota axis orchestrates gut homeostasis in a diarrheal pig model. Microbiome. 2022;10:139. 10.1186/s40168-022-01326-8. 10.1186/s40168-022-01326-8 PMC9429786 36045454

[23] Venkatakrishnan V, Quintana-Hayashi MP, Mahn M, Haesebrouck F, Pasman F, Lindén SK. Infection regulates mucin glycosylation synthesis inducing an increased expression of core-2 -glycans in porcine colon. J Proteome Res. 2017;16(4):1728–42. 10.1021/acs.jproteome.7b00002. 28301166 10.1021/acs.jproteome.7b00002

[24] Ni J, Wu GD, Albenberg L, Tomov VT. Gut microbiota and IBD: causation or correlation. Nat Rev Gastroenterol Hepatol. 2017;14(10):573–84. 10.1038/nrgastro.2017.88. 28743984 10.1038/nrgastro.2017.88 PMC5880536

[25] Pickard JM, Zeng MY, Caruso R, Núñez G. Gut microbiota: role in pathogen colonization, immune responses, and inflammatory disease. Immunol Rev. 2017;279(1):70–89. 10.1111/imr.12567. 28856738 10.1111/imr.12567 PMC5657496

[26] Faye AS, Allin KH, Iversen AT, Agrawal M, Faith J, Colombel JF, et al. Antibiotic use as a risk factor for inflammatory bowel disease across the ages: a population-based cohort study. Gut. 2023;72(4):663–70. 10.1136/gutjnl-2022-327845. 36623926 10.1136/gutjnl-2022-327845 PMC9998355

[27] Al Mijan M, Lim BO. Diets, functional foods, and nutraceuticals as alternative therapies for inflammatory bowel disease: present status and future trends. World J Gastroenterol. 2018;24(25):2673–85. 10.3748/wjg.v24.i25.2673. 29991873 10.3748/wjg.v24.i25.2673 PMC6034142

[28] Britto S, Kellermayer R. Carbohydrate monotony as protection and treatment for inflammatory bowel disease. J Crohns Colitis. 2019;13(7):942–8. 10.1093/ecco-jcc/jjz011. 30715243 10.1093/ecco-jcc/jjz011

[29] Che TM, Johnson RW, Kelley KW, Van Alstine WG, Dawson KA, Moran CA, et al. Mannan oligosaccharide improves immune responses and growth efficiency of nursery pigs experimentally infected with porcine reproductive and respiratory syndrome virus. J Anim Sci. 2011;89(8):2592–602. 10.2527/jas.2010-3208. 21454863 10.2527/jas.2010-3208

[30] Shen YB, Piao XS, Kim SW, Wang L, Liu P, Yoon I, et al. Effects of yeast culture supplementation on growth performance, intestinal health, and immune response of nursery pigs. J Anim Sci. 2009;87(8):2614–24. 10.2527/jas.2008-1512. 19395514 10.2527/jas.2008-1512

[31] Poeikhampha T, Bunchasak C. Comparative effects of sodium gluconate, mannan oligosaccharide and potassium diformate on growth performances and small intestinal morphology of nursery pigs. Asian Australas J Anim Sci. 2021;24(6):844–50. 10.5713/ajas.2011.10334.

[32] Gao H, Sun FZ, Lin G, Guo YH, Zhao JB. Molecular actions of different functional oligosaccharides on intestinal integrity, immune function and microbial community in weanling pigs. Food Funct. 2022;13(23):12303–15. 10.1039/D2FO02243E. 36349889 10.1039/d2fo02243e

[33] Spring P, Wenk C, Connolly A, Kiers A. A review of 733 published trials on Bio-Mos®, a mannan oligosaccharide, and Actigen®, a second generation mannose rich fraction, on farm and companion animals. J Appl Anim Nutr. 2015;3:e8. 10.1017/jan.2015.6.

[34] Teng PY, Kim WK. Review: roles of prebiotics in intestinal ecosystem of broilers. Front Vet Sci. 2018. 10.3389/fvets.2018.00245. 30425993 10.3389/fvets.2018.00245 PMC6218609

[35] Cheng WW, Lu J, Li BX, Lin WS, Zhang Z, Wei X, et al. Effect of functional oligosaccharides and ordinary dietary fiber on intestinal microbiota diversity. Front Microbiol. 2017. 10.3389/fmicb.2017.01750. 28979240 10.3389/fmicb.2017.01750 PMC5611707

[36] Yu E, Chen DW, Yu B, Huang ZQ, Mao XB, Zheng P, et al. Manno-oligosaccharide attenuates inflammation and intestinal epithelium injury in weaned pigs upon enterotoxigenic *Escherichia coli* K88 challenge. Brit J Nutr. 2021;126(7):993–1002. 10.1017/S0007114520004948. 33298213 10.1017/S0007114520004948

[37] Rouissi A, Alfonso-Avila AR, Guay F, Boulianne M, Létourneau-Montminy MP. Effects of *Bacillus subtilis*, butyrate, mannan-oligosaccharide, and naked oat (β-glucans) on growth performance, serum parameters, and gut health of broiler chickens. Poultry Sci. 2021;100(12):101506. 10.1016/j.psj.2021.101506. 10.1016/j.psj.2021.101506 PMC8571078 34731741

[38] Kogut MH, Arsenault RJ. Gut Health: The new paradigm in food animal production. Front Vet Sci. 2016. 10.3389/fvets.2016.00071. 10.3389/fvets.2016.00071 PMC5005397 27630994

[39] Johansson MEV, Phillipson M, Petersson J, Velcich A, Holm L, Hansson GC. The inner of the two Muc2 mucin-dependent mucus layers in colon is devoid of bacteria. Proc Natl Acad Sci U S A. 2008;105(39):15064–9. 10.1073/pnas.0803124105. 18806221 10.1073/pnas.0803124105 PMC2567493

[40] Celi P, Cowieson AJ, Fru-Nji F, Steinert RE, Kluenter AM, Verlhac V. Gastrointestinal functionality in animal nutrition and health: new opportunities for sustainable animal production. Anim Feed Sci Technol. 2017;234:88–100. 10.1016/j.anifeedsci.2017.09.012.

[41] Bergstrom KSB, Xia LJ. Mucin-type -glycans and their roles in intestinal homeostasis. Glycobiology. 2013;23(9):1026–37. 10.1093/glycob/cwt045. 23752712 10.1093/glycob/cwt045 PMC3858029

[42] Burger-van Paassen N, Vincent A, Puiman PJ, van der Sluis M, Bouma J, Boehm G, et al. The regulation of intestinal mucin MUC2 expression by short-chain fatty acids: implications for epithelial protection. Biochem J. 2009;420:211–9. 10.1042/BJ20082222. 19228118 10.1042/BJ20082222

[43] González-Morelo KJ, Vega-Sagardía M, Garrido D. Molecular insights into -linked glycan utilization by gut microbes. Front Microbiol. 2020. 10.3389/fmicb.2020.591568. 10.3389/fmicb.2020.591568 PMC7674204 33224127

[44] Tong M, McHardy I, Ruegger P, Goudarzi M, Kashyap PC, Haritunians T, et al. Reprograming of gut microbiome energy metabolism by the Crohn’s disease risk polymorphism. Isme J. 2014;8(11):2193–206. 10.1038/ismej.2014.64. 24781901 10.1038/ismej.2014.64 PMC4992076

[45] Jacobs JP, Lin L, Goudarzi M, Ruegger P, McGovern DPB, Fornace AJ, et al. Microbial, metabolomic, and immunologic dynamics in a relapsing genetic mouse model of colitis induced by T-synthase deficiency. Gut Microbes. 2017;8(1):1–16. 10.1080/19490976.2016.1257469. 27874308 10.1080/19490976.2016.1257469 PMC5341916

[46] Kim CH, Kim D, Ha Y, Cho KD, Lee BH, Seo IW, et al. Expression of mucins and trefoil factor family protein-1 in the colon of pigs naturally infected with *Salmonella typhimurium*. J Comp Pathol. 2009;140(1):38–42. 10.1016/j.jcpa.2008.10.002. 19064270 10.1016/j.jcpa.2008.10.002

[47] Qu DW, Wang G, Yu LL, Tian FW, Chen W, Zhai QX. The effects of diet and gut microbiota on the regulation of intestinal mucin glycosylation. Carbohyd Polym. 2021. 10.1016/j.carbpol.2021.117651. 10.1016/j.carbpol.2021.117651 33593539

[48] Kelm M, Quiros M, Azcutia V, Boerner K, Cummings RD, Nusrat A, et al. Targeting epithelium-expressed sialyl Lewis glycans improves colonic mucosal wound healing and protects against colitis. JCI Insight. 2020. 10.1172/jci.insight.135843. 32427587 10.1172/jci.insight.135843 PMC7406298

[49] Kudelka MR, Stowell SR, Cummings RD, Neish AS. Intestinal epithelial glycosylation in homeostasis and gut microbiota interactions in IBD. Nat Rev Gastroenterol Hepatol. 2020;17(10):597–617. 10.1038/s41575-020-0331-7. 32710014 10.1038/s41575-020-0331-7 PMC8211394

[50] Zhang X, Wang C, Han Q, Chen X, Li GY, Yu GL. Highly sialylated mucin-type glycopeptide from porcine intestinal mucosa after heparin extraction: O-glycan profiling and immunological activity evaluation. Glycoconj J. 2021;38(5):527–37. 10.1007/s10719-021-10014-y. 34480673 10.1007/s10719-021-10014-y

[51] Moran AP, Gupta A, Joshi L. Sweet-talk: role of host glycosylation in bacterial pathogenesis of the gastrointestinal tract. Gut. 2011;60(10):1412–25. 10.1136/gut.2010.212704. 21228430 10.1136/gut.2010.212704

[52] Valdés-Ramos R, Martínez-Carrillo BE, Aranda-González II, Guadarrama-López AL, Jarillo RA. Moderate exercise and its effect on the systemic immune response associated to high fat or high carbohydrate diets. Faseb J. 2010. 10.1096/fasebj.24.1_supplement.723.12.

[53] Rao CV, Janakiram NB, Madka V, Kumar G, Scott EJ, Pathuri G, et al. Small-molecule inhibition of GCNT3 disrupts mucin biosynthesis and malignant cellular behaviors in pancreatic cancer. Cancer Res. 2016;76(7):1965–74. 10.1158/0008-5472.CAN-15-2820. 26880801 10.1158/0008-5472.CAN-15-2820

[54] Li CW, Lim SO, Chung EM, Kim YS, Park AH, Yao J, et al. Eradication of triple-negative breast cancer cells by targeting glycosylated PD-L1. Cancer Cell. 2018;33(2):187. 10.1016/j.ccell.2018.01.009. 29438695 10.1016/j.ccell.2018.01.009 PMC5824730

