# Supplementary material for: Mannooligosaccharides modulate mucin O-glycan–gut microbe interactions and preserve the intestinal barrier in weaned piglets
Source: J Anim Sci Biotechnol. 2026 Aug 24;17:175. doi: 10.1186/s40104-026-01492-x (PMC13501716; doi:10.1186/s40104-026-01492-x)
Supplement: Supplementary file 1 [file 40104_2026_1492_MOESM1_ESM.docx]

Table S1 Composition and nutrient levels of the experimental diet (%, as-fed basis)

| Items | Content (%) |
| --- | --- |
| Ingredients |  |
| Corn | 52.53 |
| Soybean meal | 19.00 |
| Full-fat soybean powder | 9.00 |
| Fish meal | 4.00 |
| Whey powder | 8.00 |
| Soybean oil | 3.60 |
| Dicalcium phosphate | 0.90 |
| L-Lysine-HCl, 78% | 0.46 |
| L-Threonine | 0.15 |
| DL-Methionine | 0.10 |
| L-Tryptophan | 0.03 |
| L-Valine | 0.12 |
| Salt | 0.30 |
| Limestone | 0.81 |
| Premix^a^ | 1.00 |
| Total | 100.00 |
| Calculated nutrients |  |
| Digestible energy（MJ/kg） | 14.76 |
| Crude protein | 19.38 |
| Lysine | 1.35 |
| Methionine | 0.39 |
| Threonine | 0.79 |
| Calcium | 0.80 |
| Total phosphorus | 0.62 |

^a^The premix provided the following (per kilogram of complex feed): Vitamin A,12,000 IU; Vitamin D, 2,500 IU; Vitamin E, 30 IU; Vitamin B12, 12µg; Vitamin K,3 mg; d-pantothenic acid, 15 mg; nicotinic acid, 40 mg; choline chloride, 400 mg;Mn, 40 mg; Zn, 100 mg; Fe, 90 mg; Cu, 8.8 mg; I, 0.35 mg; Se, 0.3 mg.

Table S2 Primer sequences information

| Target genes | Primer forward/reverse | Primer sequence (5’→3’) |
| --- | --- | --- |
| β-actin | Forward | CTGCGGCATCCACGAAACT |
|  | Reverse | AGGGCCGTGATCTCCTTCTG |
| MUC2 | Forward | GTCGAGTACATCCTGCTGACG |
|  | Reverse | GAGTCCTCTCTGTTCCACACG |
| MUC5AC | Forward | CAGACGGTCATCGTGGTCAA |
|  | Reverse | TGAAGAGCATGACGTTGGCT |
| MUC5B | Forward | GAGCTCTCCAACGGCTCC |
|  | Reverse | GTAGGGCAGCTCCTCCCTCT |

Table S3 Taxa altered in the colon of piglets

| Taxonomic name | group | LDA values | *P* value |
| --- | --- | --- | --- |
| d__Bacteria.p__Firmicutes.c__Bacilli.o__Erysipelotrichales.f__Erysipelatoclostridiaceae.g__UCG_004 | CON | 4.24054 | 0.02222 |
| d__Bacteria.p__Firmicutes.c__Bacilli.o__Erysipelotrichales.f__Erysipelotrichaceae.g__Turicibacter | CON | 3.81340 | 0.01448 |
| d__Bacteria.p__Firmicutes.c__Clostridia.o__Oscillospirales.f__Ruminococcaceae.g___Eubacterium__siraeum_group | CON | 3.72512 | 0.02472 |
| d__Bacteria.p__Proteobacteria.c__Gammaproteobacteria.o__Enterobacterales | CON | 3.87084 | 0.02497 |
| d__Bacteria.p__Proteobacteria.c__Gammaproteobacteria.o__Enterobacterales.f__Enterobacteriaceae | CON | 3.87084 | 0.02497 |
| d__Bacteria.p__Proteobacteria.c__Gammaproteobacteria.o__Enterobacterales.f__Enterobacteriaceae.g__Escherichia_Shigella | CON | 3.87084 | 0.02497 |
| d__Bacteria.p__Proteobacteria.c__Gammaproteobacteria.o__Pasteurellales | CON | 3.57638 | 0.02223 |
| d__Bacteria.p__Proteobacteria.c__Gammaproteobacteria.o__Pasteurellales.f__Pasteurellaceae | CON | 3.57641 | 0.02223 |
| d__Bacteria.p__Proteobacteria.c__Gammaproteobacteria.o__Pasteurellales.f__Pasteurellaceae.g__Actinobacillus | CON | 3.58709 | 0.02223 |
| d__Bacteria.p__Firmicutes.c__Bacilli | MOS | 4.90785 | 0.01041 |
| d__Bacteria.p__Firmicutes.c__Bacilli.o__Erysipelotrichales.f__Erysipelotrichaceae.g___Anaerorhabdus__furcosa_group | MOS | 4.35790 | 0.03089 |
| d__Bacteria.p__Firmicutes.c__Bacilli.o__Lactobacillales | MOS | 4.91415 | 0.01041 |
| d__Bacteria.p__Firmicutes.c__Bacilli.o__Lactobacillales.f__Lactobacillaceae | MOS | 4.92386 | 0.00395 |
| d__Bacteria.p__Firmicutes.c__Bacilli.o__Lactobacillales.f__Lactobacillaceae.g__Lactobacillus | MOS | 4.92386 | 0.00395 |
